# Social Isolation and Loneliness I Cognitive and Psychosocial Barriers to Social Engagement in Older Adults

**DOI:** 10.1093/geroni/igaf122.2776

**Published:** 2025-12-31

**Authors:** Molly Split, Sarah Prieto, Zachary Kunicki, Alyssa De Vito, Megan Barker, Louisa Thompson, Kathryn Devlin, Maria Schultheis

**Affiliations:** Brown University, Providence, Rhode Island, United States; Brown University, Providence, Rhode Island, United States; Warren Alpert Medical of Brown University, Providence, Rhode Island, United States; Brown University, Providence, Rhode Island, United States; Brown University, Providence, Rhode Island, United States; Warren Alpert Medical of Brown University, Providence, Rhode Island, United States; Drexel University, Philadelphia, Pennsylvania, United States; Drexel University, Philadelphia, Pennsylvania, United States

## Abstract

Loneliness in older adulthood can have profound effects on cognition and quality of life. While increased social engagement can alleviate loneliness, interventions to improve social connections remain limited. Developing effective interventions requires a deeper understanding of personal barriers and cognitive difficulties that may impede social engagement. We investigated these factors in a survey with cognitively healthy older adults. Participants were 167 racially and ethnically diverse older adults (M age=60.4, M education=16.1, 56% women). Participants completed a survey developed by our research team on social engagement frequency and barriers, and social life satisfaction. Online cognitive assessments of attention, working memory, and response inhibition were also administered. Spearman rank-order correlations examined associations between these measures. Common barriers to socialization included changes in interests (53%) and mood (41%). Lower social engagement was associated with more barriers (r=-.22, p=.005) and lower social life satisfaction (r=.52, p<.001), and more barriers correlated with lower social life satisfaction (r=-.30, p<.001). Worse attention (r=-.39, p<.001), working memory (r=-.18, p=.020), and response inhibition (r=-.30, p<.001) were linked to more barriers but not social engagement frequency or satisfaction. These findings highlight the need for interventions that address barriers to social engagement, such as changes in interests and mood, as well as cognitive difficulties that may limit participation in social activities. By addressing psychological and cognitive factors, tailored interventions can be developed to promote meaningful social connections and improve well-being in aging populations.

